# Combination therapy with oncolytic viruses for lung cancer treatment

**DOI:** 10.3389/fonc.2025.1524079

**Published:** 2025-04-03

**Authors:** Lei Sun, Qi Zhao, Liyun Miao

**Affiliations:** ^1^ Department of Pharmacy, Yancheng Branch of Nanjing Drum Tower Hospital, Yancheng, Jiangsu, China; ^2^ Department of Respiratory and Critical Care Medicine, Nanjing Drum Tower Hospital, Nanjing, Jiangsu, China

**Keywords:** oncolytic viruses (OVs), combination therapy, lung cancer, combined immunization, OVs tumor vaccine

## Abstract

Lung cancer is the leading cause of cancer-related death globally. Despite various treatment options, adverse reactions and treatment resistance limit their clinical application and efficacy, therefore, new effective treatment options are still needed. Oncolytic viruses (OVs) are a new anti-cancer option. With a powerful anti-tumor effect, OVs are gradually being applied to the treatment of solid tumor. In clinical practice, we have found that in patients with NSCLC and SCLC, OVs combined with immune checkpoint inhibitors (ICI) treatment make tumor with poor response to immunotherapy become sensitive. Furthermore, studies have shown that OVs combined with chemotherapy, radiation therapy, and other immune approaches (such as anti-pd1 drugs) have synergistic effects. These studies suggest that OVs combined therapy may bring hope for the treatment of lung cancer patients. This article will review the current status and prospect of OVs combination therapy in the field of lung cancer treatment and summarizes the mechanism of action.

## 1 Introduction

Lung cancer is one of the most common and deadly cancers in the world (1, 2). Globally, with around 2 million new cases and 1.76 million deaths each year, this has become a major public health problem (1). The clinical pathological types are mainly non-small cell lung cancer (NSCLC) and small cell lung cancer (SCLC) (3). The clinical treatment of lung cancer has always been a hot and difficult topic in the medical field (4). Although the traditional treatment methods such as surgery, chemotherapy and radiotherapy have certain effects, the treatment effect of advanced or metastatic lung cancer is still unsatisfactory, and there are many side effects and tolerance problems (5). Immunotherapy and targeted therapy have become common methods of lung cancer treatment, which have greatly improved patient survival (5–7). However, with the progress of treatment, the problem of drug resistance is inevitable, and patients with drug-resistant lung cancer usually progress rapidly and have poor long-term survival (8). In general, although there are many drugs for the treatment of lung cancer, the complexity and adaptability of the tumor limit the therapeutic effect, and it is urgent to explore new therapeutic ideas.

Oncolytic viruses (OVs) can directly dissolve tumor cells and reshape the tumor microenvironment, providing new options for cancer therapy (9–14). In recent years, OVs have gradually attracted people’s attention because of its effectiveness in the treatment of advanced solid tumors (15, 16). Combination therapy strategies help to improve antitumor outcome. It has been found that the combination of paclitaxel and a novel replicable recombinant oncolytic herpes simplex virus type 1(VG161) can induce proinflammatory changes in the tumor microenvironment and reduce breast cancer pulmonary metastasis (17). The combined oncolytic influenza A viruses (IAVs) and immune checkpoint inhibition (ICI) therapy can result in a sustained anti-tumor efficacy against metastatic pulmonary melanoma (18). A phaseIIrandomized trial evaluated the combination of OV plus ICI versus ICI alone in patients with advanced melanoma, and the results indicated that the combination has greater antitumor activity without additional safety concerns versus ICI alone (19). These studies suggest that OVs combination therapy has great potential in the clinical treatment of cancer.

In the treatment of lung cancer, the combination of other drugs is also being explored to improve efficacy. The combination of OVs with other therapies (such as ICIs, chemotherapy or radiotherapy) play a synergistic anti-tumor role (20–23). Clinical application is still in the exploratory stage. A phase II study has explored the combination of TG4010 with first line chemotherapy in patients with stage IIIB/IV NSCLC, the results showed the combination regimen is feasible and one complete and one partial response were observed out of 14 evaluable patients, and it seemed to improve progression-free survival relative to placebo plus chemotherapy (24, 25). In our previous study, we reported that a case of OVs (intratumoral injection of recombinant human adenovirus type 5) combined with nivolumab treated a patient with ICI resistant NSCLC after nivolumab treatment, effectively improving the patient’s condition and achieving disease control (26). This is the first report to describe the reversal of immune resistance by oncolytic viruses in non-small cell lung cancer, providing a treatment option for ICI resistant patients. Subsequently, our team applied recombinant human adenovirus type 5 to a patient with SCLC, successfully reversed the patient’s resistance to ICI, and applied multiple fluorescent immunohistochemical technology to detect the changes in the patient’s immune microenvironment before and after oncolytic virus treatment (27). We found that immune cell infiltration decreased after the first injection and increased during subsequent injections. After 5 months of combined treatment with oncolytic viruses, CD8+ T cell infiltration appeared to be more complete. The results suggest that oncolytic virus therapy combined with immune checkpoint inhibitors seems to activate anti-tumor immunity, suggesting the feasibility of its clinical application, but more evidence is lacking.

Overall, the application of OVs therapy in the treatment of lung cancer is still at an early stage and cannot provide strong clinical evidence. There are many attempts of OVs combination therapy, and new studies are emerging constantly, lacking of systematic sorting. Therefore, this paper summarized the current studies, comprehensively discussed the molecular mechanism and clinical application of the combination therapy regimen, and provided references for subsequent studies.

## 2 Mechanism of action of OVs

### 2.1 Direct cytolytic activity and induction of systemic anti-tumor response

OVs are able to selectively recognize and infect tumor cells, replicating in large numbers within the cells and ultimately leading to the lysis and death of the tumor cells (9, 12). Masemann, D et al. explored oncolytic properties of IAVs infection against NSCLCs in an immunocompetent model *in vivo*, and results showed that infection with low-pathogenic IAV leads to rapid and efficient oncolysis, eliminating 70% of the initial tumor mass (28). IAV infection caused a functional reversion of immunosuppressed tumor-associated lung macrophages into a M1-like pro-inflammatory active phenotype that additionally supported virus-induced death of cancer cells (28). Ye, T et al. indicated that oncolytic Newcastle disease virus (NDV) is a potent immunogenic cell death inducer and that autophagy contributes to this process in lung cancer cells (29). They used Annexin V and propyl iodide (PI) double staining and flow cytometry to detect the apoptosis of lung cancer cells 24 h and 48 h after NDV infection. Activation of caspase-3 and cleaved poly (ADP-ribose) polymerase (PARP) was detected by western blot analysis. It was clearly confirmed that NDV influences the process of inducing apoptosis, but the related pathways are still being explored (29).

**Figure 1:**
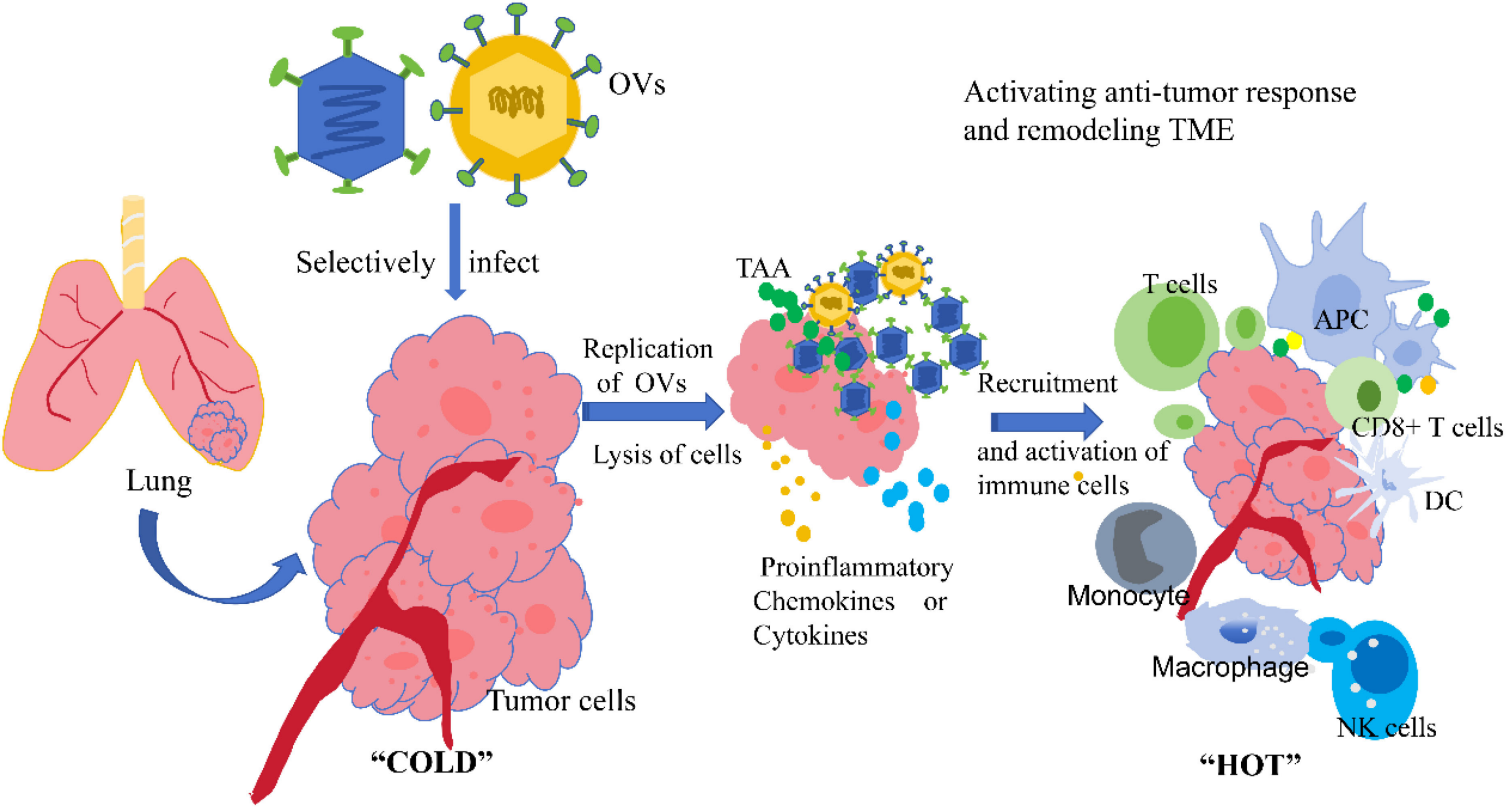
Mechanism of anti-tumor action of oncolytic virus. OVs, oncolytic viruses; TAA, tumor-associated antigens; APC, antigen-presenting cells; DC, dendritic cell; NK, natural killer cell; TME, tumor microenvironment. Direct Oncolysis: OVs can selectively recognize and infect tumor cells, replicate extensively within them, and eventually cause tumor cell lysis and death; Release of Tumor Antigens: After tumor cells are lysed by oncolytic viruses, they release substances such as TAA, which can be taken up, processed, and presented by antigen-presenting cells (e.g., DC); Recruitment and Activation of Immune Cells: During the infection of tumor cells by oncolytic viruses, pro-inflammatory cytokines and chemokines are induced in the tumor microenvironment, recruiting and activating immune cells such as T cells, NK cells, and macrophages. Activated CD8+ T cells and other immune cells can specifically recognize and kill tumor cells, while also transforming the tumor microenvironment from an immunosuppressive “cold” state to an immunostimulatory “hot” state, thereby enhancing the overall anti-tumor immune response of the body.

On the one hand, the lysis of tumor cells by viruses releases large amounts of tumor-associated antigens, which can activate the immune system of the body and promote the uptake and processing of antigens by antigen-presenting cells such as dendritic cells. This can then activate immune cells such as T cells to produce specific anti-tumor immune responses (11, 30, 31). On the other hand, oncolytic viruses can also regulate immunosuppressive factors in the tumor microenvironment, such as reducing the number or inhibiting the function of regulatory T cells, to enhance the cytotoxicity of immune cells against tumor cells (11, 32). This immune modulation effect can not only directly kill tumor cells but also produce an immune memory effect, making the body generate long-term immune surveillance against tumor cells and preventing the recurrence and metastasis of cancer (13, 33). The anti-tumor mechanism of OV is shown in the **Figure 1**.

### 2.2 The role of the tumor microenvironment (TME) in making lung cancer susceptible to OVs therapy

The tumor microenvironment is complex, including immune cells, blood vessels, extracellular matrix (ECM), etc., which work together to affect tumor growth, metastasis and resistance (34).

Regulation of immune cells in TME: In lung cancer, NK cells and cytotoxic T cells are typically reduced, while myeloid-derived suppressive cells and regulatory T cells are increased. Macrophages and neutrophils may also transform from pro-inflammatory to pro-tumorigenic phenotypes (35). OVs therapy can alter this imbalance of immune cells, increasing the number and activity of immune cells such as cytotoxic T cells, thereby enhancing the killing of tumor cells (36, 37).

The role of ECM: It can influence the infection and spread of OVs in tumors. After OVs treatment, the composition and structure of the extracellular matrix may be changed to reduce its obstruction to the spread of the virus, so that the virus can infect tumor cells more widely and play oncolytic role (38).

Regulation of immunosuppressive factors: OVs therapy can regulate the expression of immunosuppressive factors, such as inducing tumor cells to produce cytokines such as interferon, thereby downregulating the expression of PD-L1, enhancing the recognition and killing of tumor cells by immune cells, and improving the sensitivity of lung cancer to OVs therapy (39). This process is mainly achieved by affecting IFN, significantly induces the expansion of activated tumor-infiltrating lymphocytes, including IFN-γ^+^CD8^+^ T cells, IFN-γ^+^TNF-α^+^CD8^+^ T cells, and GZMB^+^CD8^+^ T cells. It can revitalize tumor-infiltrating CD8^+^ T cells and up-regulate the expression of CTLA-4 and TIM-3 on depleted CD8^+^ T cells, resulting in high levels of CTLA-4^+^ Treg cells (39).

Despite the fact that OV can directly kill some tumor cells and induce anti-tumor immunity, the effect of OV alone in tumor treatment is limited. The extracellular matrix (ECM) in the TME can impede the spread of oncolytic viruses. ECM is composed of various cellular components, including collagen and fibronectin, and in tumors, it may become dense, limiting the spread of oncolytic viruses in tumor tissue (40). Furthermore, repeated oncolytic virus therapy may lead to the production of antibodies against OV and a series of adaptive changes by the immune system. All of these limitations restrict the clinical effect of OV. Combining OV with other anti-tumor therapies may improve this situation and exert synergistic effects to combat tumor resistance and achieve better clinical outcomes.

## 3 Mechanism of combined oncolytic therapy in lung cancer

### 3.1 Combined with chemotherapy

When combined with OVs, chemotherapy drugs can enhance the infectivity of OVs to tumor cells, for example, by altering the permeability of the tumor cell membrane or metabolic state, making the virus easier to enter the cell. At the same time, the immune response triggered by oncolytic viruses can enhance the anti-tumor effects of chemotherapy drugs, overcoming tumor cell resistance to chemotherapy drugs (41, 42).

Tokunaga, Y constructed an adenoviral vector that encoded a short hairpin siRNA targeting the RRM1 gene (Ad-shRRM1) that increased sensitivity to gemcitabine of each type of RRM1-overexpressing tumor cell NSCLC lines and human tumor xenograft model in nude mice. Results showed that combination with Ad-shRRM1 and gemcitabine exerted significantly greater inhibition on cell proliferation than Ad-shRRM1 or gemcitabine alone. It may be a treatment option for patients with NSCLC (21).

Gomez-Gutierrez et al. evaluated the therapeutic potential of oncolytic adenovirus and temozolomide (TMZ) *in vitro* and *in vivo* treatment of lung cancer cells and found that both synergistic enhancement of cancer cell death; They propose three related mechanisms: apoptosis, viral replication, and autophagy. Autophagy inhibition partially protects cancer cells from this combination therapy. The combination significantly inhibited the growth of subcutaneous H441 lung cancer xenografts in naked mice (20). Garofalo et al. found that chemotherapy drugs combined with OVs showed stronger cytotoxic reaction and oncolytic effect. They discovered that the whole-body delivery of oncolytic adenovirus and paclitaxel encapsulated in extracellular vesicle (EV) formulation, *in vitro*, significantly improved transduction and infection titer compared to the virus and paclitaxel alone, and had enhanced anti-cancer effects in both *in vitro* and *in vivo* lung cancer models (23). Kellish, P et al. found that a modified oncolytic modified oncolytic myxoma virus (MYXV) combined with low-dose cisplatin improved survival of immunocompetent SCLC mice model (43). These work offers a strategy for combining chemotherapy drugs and viral therapies as a treatment for lung cancer.

### 3.2 Combined with immunotherapy

Preclinical studies suggest that oncolytic virus therapy can increase tumor immunogenicity, reshape immunosuppressive TME, and lead to increased antitumor response to immunotherapy drugs (14, 44). At present, the synergistic effect of oncolytic viruses combined with immunotherapy has been reported in many tumor species, such as prostate tumors (45), glioblastoma (32), breast cancer (17, 46). The mechanism of oncolytic virus combined immunotherapy in the treatment of lung cancer is as follows:

#### 3.2.1 OVs with ICIs

Dorthe Masemann et al. found the synergistic antitumor effect of oncolytic influenza virus and B7-H3 immune junction inhibitor (monoclonal alpha-B7-H3 antibody (Enoxumab)) on ICI resistant lung cancer through experimental studies. They combined oncolytic cell IAV-infection and ICIs, two anti-cancer methods, for treatment. The results showed that IAV infection combined with novel B7-H3 ICI resulted in increased levels of M1-polarized alveolar macrophages and increased cytotoxic lymphocyte infiltration, ultimately significantly improving the oncolytic performance of about 80% of existing tumors. In contrast, the application of clinically approved alpha-PD-1 IC antibodies alone or in combination with oncolytic cell IAV did not provide additional oncolytic or immunomodulatory benefits. The study suggests that individualized therapy using synergistic oncolytic cells IAV and B7-H3 ICI may be a viable approach to target patients with non-small cell lung cancer resistant to approved ICIs (47).

Sun, F et al. established a urethane induced mouse endogenous lung tumor model similar to human lung cancer, in which intravenous oncolytic vaccinia virus (oVV) was synergistic with simultaneous, rather than single, blocking of PD-1 and t cell immunoglobulin and mucin-domain containing-3 (TIM-3). In addition to directly killing tumor cells, oVV also induces lung recruitment of T cells, tumor invasion, and expression of PD-1 and TIM-3 ligands on T cells, and PD-1 and TIM-3 ligands on tumor cells and tumor-associated immune cells. Blocking PD-1 or TIM-3 also causes their mutual induction of T cells. Their findings suggest that triple therapy is more effective for refractory lung cancers (48).

Siarhei Sitnik et al. found that infection with oncolytic IAV in primary lung tumor-bearing mice resulted in strong virus-induced tumor cell lysis and restoration of innate immune cell immunity. This study analyzed the oncolytic and immunomodulatory efficiency of IAV *in vivo* against lung B16-F10 lung metastasis and found that IAV infection eliminated melanoma-mediated lung immunosuppression and induced more than 50% of cancer cell lysis. Oncolytic effects reached maximum efficacy 3 days after infection, but did not persist over time. To maintain virus-induced antitumor effects, in addition to influenza virus infection, they combined an immune checkpoint inhibitor targeting PD-1 receptor to treat melanogen-derived lung cancer in mice. The results showed that the combination of IAV and ICI produced a sustained antitumor effect, with lung melanoma masses still reduced by 50% on day 12 of IAV infection compared with control mice. The study showed that ICI treatment strongly enhanced the oncolytic effect of influenza virus infection, suggesting that combination therapy is a promising approach for the treatment of metastatic pulmonary melanoma (18).

#### 3.2.2 With other immunotherapy drugs

NG-347 is Tumor-Specific Immuno-Gene (T-SIGn) virus, expressing chemokines and immune stimulating transgenes, which encodes IFNα, MIP1α and CD80. The study by Sonzogni, O, et, al. showed that T-SIGn tumor reengineering therapy and CAR T cells synergize to clear A549 human tumor xenografts and their pulmonary metastases in NSG mice bearing human tumor xenografts. The study showed that NG-347 reprogramed the TME toward a pro-inflammatory state, which resulted in the recruitment and activation of both CAR T cells and mouse innate immune cells (49).

### 3.3 With molecular targeted drugs

Both NSCLC and SCLC cells express receptor tyrosine kinases, which may be overexpressed or mutated in lung cancer, leading to increased activation. The c-Met receptor tyrosine kinase is crucial for cell transformation and tumor growth, invasion.

Zhou, X. et, al. discovered that MEK Inhibitor (MEKi) Trametinib can enhance therapeutic efficacy of Oncolytic Herpes Simplex Virus (oHSV) in some Lung carcinoma models. They investigated the oncolytic activity *in vitro* and anti-tumor therapeutic efficacy *in vivo* when combined with oHSV and Trametinib. They found that (1): Trametinib augmented oHSV oncolytic activity in BRAF V600E-mutated tumor cells (2). Combination treatment with oHSV and Trametinib enhanced virus replication mediated by down-regulation of STAT1 and PKR expression or phosphorylation in BRAF V600E-mutated tumor cells as well as BRAF wt/KRAS-mutated tumor cells (3). when combined oHSV with PD-1 blockade and MEK inhibition, they found a remarkably synergistic therapeutic efficacy *in vivo* for BRAF wt/KRAS-mutated tumor models. Collectively, their data provide some evidence for clinical application of combination therapy with oncolytic virus, MEK inhibition, and checkpoint blockade for BRAF or KRAS-mutated tumors (50). The study by Cui, B, et al., found a synergistic effect of oncolytic coxsackievirus B5/Faulkner (CV-B5/F) and the inhibitors of DNA-dependent protein kinase (DNA-PK) or ataxia telangiectasia mutated protein (ATM) on NSCLCs through inducing apoptosis and autophagy. In this study, they constructed a PDX model by transplanting patient-derived NSCLC tumors into an immunocompromised mouse, providing a faithful representation of individual tumors. Then they evaluate the anticancer activity of CV-B5/DNA-PKI and CV-B5/ATMI *in vivo*, and the results showed that combination therapy elicited severe DNA damage, increased viral propagation, and insurmountable endoplasmic reticulum (ER) stress-related apoptosis and immunogenic cell death (ICD). They discovered that viral infection triggered ER stress-related pro-apoptosis and autophagy signals, whereas repair for double-stranded DNA breaks (DSBs) contributed to cell survival which can be antagonized by inhibitor-induced cell death, manifested by exacerbated DSBs, apoptosis, and autophagy. This study suggested DNA damage response (DDR) inhibitors combination with CV-B5/F is a potential therapy for NSCLCs (51). Chen, S. Y et al. explored the combination of rapamycin, a selective mTOR inhibitor with an E1B55KD-deleted, replication-selective oncolytic adenovirus (Ad.What), which is driven by the c-Met promoter, targeting lung cancer cells with c-Met overexpression. This combination increased infectivity by augmenting the expression of coxsackievirus and adenovirus receptors and αV integrin on cancer cells and induced autophagy. Results suggested that combining a c-Met promoter-driven oncolytic adenovirus with rapamycin could be an effective lung cancer treatment strategy (52). Also, there were reported synergistic effect of NF-κB signaling pathway inhibitor and oncolytic Measles Virus vaccine strain against Lung Cancer (53).

### 3.4 Combined radiotherapy

When oncolytic viruses are combined with radiotherapy, radiotherapy can increase the sensitivity of tumor cells to oncolytic viruses and promote the spread and replication of the virus in the tumor tissue. In addition, the combination of the two can enhance the immune response and produce a distant effect. Current studies have found that oncolytic herpes simplex virus therapy has a synergistic effect in the treatment of lung cancer (54).

### 3.5 Brief summary of the principle of synergies of combination therapy

**Figure 2:**
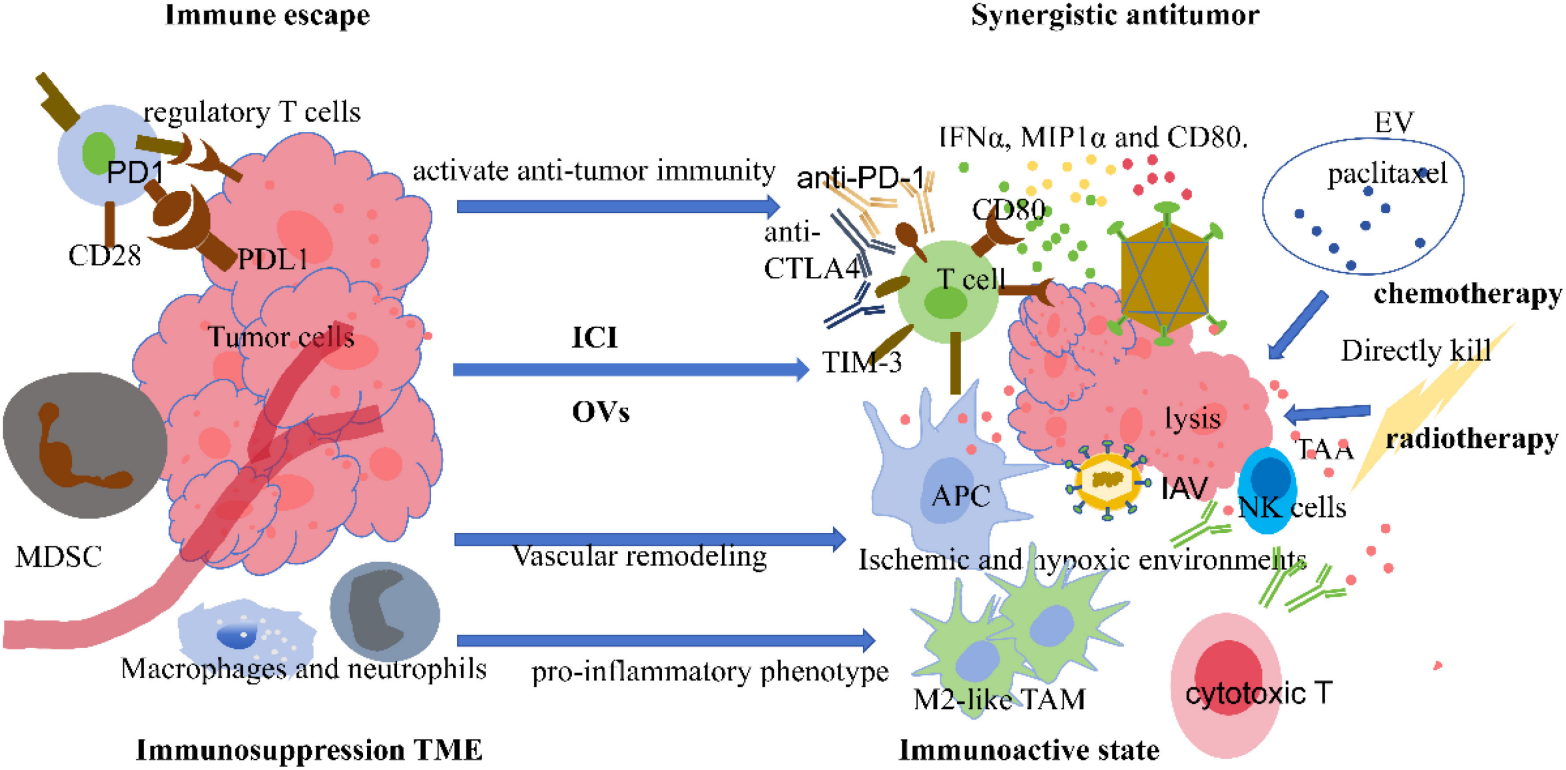
Anti-tumor mechanism of combined therapy. MDSC, myeloid-derived suppressor cells; IAV, influenza A virus; NK, natural killer cell; TAM, tumor-associated macrophages; EV, Extracellular vesicles; IFN, interferon; MIP1, Macrophage Inflammatory Protein-1; TIM-3, T cell immunoglobulin and mucin-domain containing-3. Left: Tumor immune escape and immunosuppressive microenvironment: The binding of PD-L1 on the surface of tumor cells to PD-1 on T cells, along with the presence of regulatory T cells, inhibits T cell activity, preventing them from effectively exerting anti-tumor immune responses and allowing tumor cells to escape immune surveillance. Immune cells such as M2-like TAMs have pro-inflammatory phenotypic transformation, and the tumor microenvironment features an ischemic and hypoxic environment due to vascular remodeling. These factors collectively form an immunosuppressive TME, suppressing the body’s immune function. Right: Combined anti-cancer mechanism and immune activation state: OVs can lyse tumor cells, release TAAs, and activate APCs. Concurrently, combined ICIs, such as anti-PD-1 and anti-CTLA-4, can further activate T cells and enhance anti-tumor immune responses. Activated T cells secrete IFNα, MIP1α, and other cytokines, enhancing the body’s immune activity; Exosomes carrying paclitaxel for chemotherapy can directly kill tumor cells and, in combination with oncolytic viruses, exert a synergistic anti-cancer effect; Radiotherapy can directly kill tumor cells, and the combination of oncolytic viruses and radiotherapy enhances the killing effect on tumor cells through multiple mechanisms, promoting tumor cell lysis and further activating immune NK cells, cytotoxic T cells, etc., to form an immune-activated state and synergistically combat tumors.

In lung cancer, tumor forming an immunosuppressive TME to escape immune attack, NK cells and cytotoxic T cells are usually reduced, while myeloid-derived suppressive cells and regulatory T cells are increased. Macrophages and neutrophils will transform from pro-inflammatory phenotype to a phenotype that inhibits the immune response and favors tumor growth (35). OVs therapy can change the imbalanced state of immune cells and increase the number and activity of immune cells. Combining immune therapy drugs (such as ICI) can release the inhibitory state of immune cells, allowing immune cells that have been activated by OVs to more efficiently kill tumor cells (55). At the same time, OVs therapy can regulate the expression of immune inhibitory factors, such as inducing tumor cells to produce interferon and other cytokines to down-regulate PD-L1 expression and the inhibitory capacity of CTLA-4, enhancing the recognition and killing of tumor cells by immune cells, and improving the sensitivity of lung cancer to OVs therapy (39, 55). The two are combined to achieve a synergistic effect (47). Lung cancer itself has certain sensitivity and resistance to chemotherapy, and the combination of oncolytic virus and chemotherapy makes chemotherapy directly kill and complement the lysis of the virus, making the two combinations work together to enhance sensitization and efficacy cooperation: OVs can change the biological characteristics of tumor cells, such as affecting the permeability of the cell membrane and regulating the expression of related drug resistance proteins, making tumor cells more sensitive to chemotherapy drugs and reducing their resistance. At the same time, the damage caused by chemotherapy to tumor cells is also conducive to the better entry of OVs into tumor cells for subsequent replication and lysis activities, thereby enhancing the overall anti-tumor effect (20, 56, 57). When OVs is combined with radiation therapy, on the one hand, radiotherapy can not only directly kill tumor cells, but also induce immunogenic cell death of tumor cells, release damage related molecular and more tumor-related antigens, and activate the body’s immune system. OVs lysis of tumor cells also releases antigens, and the two cooperate to further enhance the immunogenicity of tumor cells and more fully mobilize the body’s immunity to fight against tumors (56, 58). **Figure 2** summarizes the mechanism of action of combination therapy.

## 4 Advantages, adverse effects, and comparisons of combination therapies

The advantage of combining OVs with immune therapy is that it enhances the body’s immune response and triggers a strong anti-tumor immune response, which may help overcome immune resistance. It also has strong targeting and causes fewer systemic adverse reactions. However, it also has some potential side effects: The common adverse reactions (AEs) were fatigue, chills, and neutropenia (59). Moreover, immune therapy may cause autoimmune diseases such as immune pneumonitis; combining OVs with immune therapy may cause exacerbation of inflammatory reactions and local inflammatory reactions such as arthralgia (59).

The advantage of combining chemotherapy is that chemotherapy drugs have a non-specific killing effect on tumor cells, while OVs have certain targeting (23). When used together, they can expand the scope of treatment and produce killing effects on different types of tumor cells (20). AEs are usually tolerated (59). The degree of myelosuppression still need to be concerned, and close monitoring of blood counts is needed.

When used radiotherapy with OVs together, they can combine local control with systemic treatment and improve treatment effects, reduce the recurrence and metastasis of tumors (54). There are few reports on adverse reactions when combining radiotherapy with OVs, of which radiotherapy can cause radiation inflammation.

In general, immunotherapy is a common protocol for clinical tumor treatment, and OVs combined immunization helps to improve its efficacy. For some lung cancer patients who are resistant to immunotherapy, OVs combined with immunotherapy may be an effective treatment strategy. Combined chemotherapy is mostly used for clinical treatment of tumors that are not sensitive to mutated gene detection, and combined radiotherapy is mostly used for adjuvant anti-tumor therapy after immunization or chemotherapy. At present, the choice of combination therapy is mostly based on clinical guidelines to choose immune or chemotherapy, combined with oncolytic virus, and more clinical practice results are needed to guide the selection of clinical therapy. Combination therapy can be tolerated by patients with moderate adverse effects (60). Through timely monitoring and intervention, adverse reactions can be controlled.

## 5 Application of oncolytic virus combined therapy in the clinical study of lung cancer

Different studies have adopted diverse combination therapies using oncolytic viruses. In some cases, researchers used recombinant adenovirus to treat NSCLC or SCLC patients in combination with ICI drugs, observing that the patients’ tumors were effectively controlled, tumor marker levels decreased, and the patients’ survival period was extended (26, 27). At present, there are few cases reported in the field of lung cancer combined therapy, and follow-up attempts are still to be made, to provide strong practical evidence for the application of OVs-based combination therapy in clinical lung cancer treatment.

The earliest attempt to use the combination of oncolytic viruses in the treatment of lung cancer was made by Rodryg Ramlau et al (24). They conducted a Phase II study of TG4010 in combination with chemotherapy (cisplatin and vinorelbine) in patients with stage IIIB/IV NSCLC. There were two forms of joint programs, one was direct joint programs, the other group was treated with TG4010 monotherapy until the disease progressed, and then continued with TG4010 plus cisplatin and vinorelbine. Sixty-five patients were enrolled In Arm 1, partial response was observed (13 of 44 patients, 13.5%); In Arm 2, two patients experienced stable disease for more than 6 months with TG4010 alone The median overall survival was 12.7 months (Arm 1) and 14.9 (Arm 2) One year survival rate was 53% and 60% respectively (24). This trail showed encouraging results which relieve patients’ disease, prolong OS, and is well tolerated, suggesting the combination of TG4010 with chemotherapy in advanced NSCLC is feasible. Limitations of this study include the relatively limited sample size (65 patients), which may not accurately reflect the efficacy and safety of TG4010 in a larger patient population, and the representativeness and generalization of the study results are also limited. Only 31 patients were able to evaluate the immune response, and ELISpot data could only be qualitatively analyzed, unable to accurately quantify the intensity and duration of immune response, which affected the overall evaluation of TG4010 immune activation effect. Studies select patients with positive expression of MUC1, which has a poorer prognosis, which may lead to potential negative selection bias, making study results not fully representative of all NSCLC patients. The purpose of this study is to explore two combination approaches, and without direct comparison between the two groups, it is difficult to determine which combination approach is more beneficial, and it is impossible to accurately evaluate the difference in effect of TG4010 under different combination strategies.

Elisabeth Quoix et al. investigated the efficacy and safety of TG4010 combined chemotherapy (Platinum-based chemotherapy regiments) in initially treated patients with stage IV NCSLC (NCT01383148). This phase II trail included 222 patients (TG4010 and chemotherapy 111; placebo and chemotherapy 111). In the whole population, median progression-free survival was 5.9 months in the TG4010 group and 5.1months in the placebo group (HR 0.74, p=0·019). No serious adverse events occurred in TG4010 combined with chemotherapy. The trial data showed that the combination therapy significantly improved PFS. Patients in the TG4010 combination chemotherapy group had a higher overall response rate (40% vs 29%, unilateral P=0.030), a longer duration of response (median 30.1 weeks vs 18.7 weeks), and more patients were able to receive maintenance chemotherapy. These results show that combination therapy has certain advantages in controlling tumor growth and improving patients’ quality of life. In the subgroup of patients with non-squamous tumors with baseline value of CD16, CD56, CD69 triple-positive activated lymphocytes (TrPAL) ≤Q3, combination therapy not only significantly improved progression-free survival (HR=0.59, 95% CI 0.40-0.87; P=0.0033) and significantly improved overall survival (HR=0.59, 95% CI 0.39-0.91; P=0.0072), indicating that combination therapy is more effective for specific patient groups (25). The limitation of this study is that the critical value of TrPAL detection is a key factor affecting the results of the study. In addition, the analysis of some subgroups, such as those related to PD-L1 expression, may not accurately reflect the difference in efficacy of combination therapy in patients with different levels of PD-L1 expression due to sample size limitations. This was a preliminary partial result of NCT01383148, however the trial was terminated early in the expanded trial, the later results are not reported.

Guan, J et al. reported A phase 2 study of stereotactic body radiation therapy (SBRT) and *in situ* oncolytic virus therapy in metastatic NSCLC followed by pembrolizumab (STOMP) (61). This is a single-arm, open-label phase 2 study, patients received intratumor injections of adenovirus-mediated expression of herpes simplex virus thymidine kinase (ADV/HSV-tk) and SBRT followed by pembrolizumab 200 mg IV every 3 weeks. In total 28 patients were enrolled (27 were evaluated for response). There were 2 CR (7.4%) and 7 PR (25.9%), and the ORR was 33.3%. CBR was 70.4%. Six of eight (75.0%) patients who were ICI refractory derived clinical benefits. The median PFS was 7.4 months, and median OS was 18.1 months. Only 6 (21.4%) patients had grade 3 adverse reactions or higher toxicity. The trial had an impressive response and the survival data were good In the subgroup of difficult-to-treat patients of ICI, 75.0% of patients obtained clinical benefits, indicating that the program also has a certain effect on difficult-to-treat patients, broadening the scope of treatment beneficiaries. ​The results reflect the overall effectiveness of the combination therapy in mNSCLC patients. Limitations of the study include the single-arm design and the lack of control group, which makes it impossible to directly compare the combination therapy with other therapies (such as pabolizumab alone, radiotherapy alone combined with immunotherapy, etc.), and it is difficult to determine the advantages and disadvantages of the combination therapy compared with other therapies. ​With only 28 patients included, the sample size is relatively limited and may not accurately reflect the true efficacy and safety of this treatment regimen in a large population of mNSCLC patients. Results with a small sample size may be biased, and the impact of different individual differences on the results may be amplified This study suggested that the dual approach of *in situ* ADV/HSV-tk plus valacyclovir gene therapy and SBRT to enhance the antitumor effect of pembrolizumab is a well-tolerated treatment in patients with metastatic NSCLC.

A randomized phase II trial evaluated pelareorep (a Dearing strain of reovirus serotype 3), combined with second-line chemotherapy in patients with NSCLC. In this trail, 166 patients were enrolled (14 to the safety run in). The results showed pelareorep did not improve the PFS vs. single agent chemotherapy (median PFS 3.0 months vs. 2.8 months, hazard ratio 0.90, P = 0.53), and the combination was tolerable. This experiment proves it the addition of pelareorep to chemotherapy did not improve the PFS of NSCLC patients (62).

These reported results offer new hope for immunotherapy-resistant patients with advanced lung cancer. OVs showed definite efficacy and safety in most trials, but some trials did not have a survival benefit, which may be related to specific patient populations or the choice of treatment timing. The sample size of most clinical trials is relatively small, which may make the results difficult to accurately reflect the real efficacy of OVs therapy in certain lung cancer patients. The success rate data obtained with a small sample size may have a large deviation due to individual differences and other factors, and cannot provide a solid basis for clinical application.

Similarly, differences in patient heterogeneity, tumor species, and drug selection may also lead to significant differences in response to oncolytic virus therapy across cohorts.

Therefore, we speculate that the future development direction is to further expand the sample size of the trial and design a more accurate treatment cohort: There were significant differences in curative effect between different cohorts, suggesting that more accurate screening of patients should be conducted in future studies to further explore the factors affecting curative effect and improve the pertinence of treatment. For example, differences in genetic characteristics and tumor microenvironments in different cohorts can be further analyzed to provide a strong basis for personalized treatment.

Overall, OVs combination therapy has shown potential in the treatment of lung cancer. At present, clinical trials are mainly in the stage of evaluating the efficacy and safety of OVs combined therapy for lung cancer (phase I/Phase II), and more mechanism studies and more precise clinical trial results are needed in the future to promote its clinical transformation and provide more effective treatment options for lung cancer patients.

Clinical trial recruitment for OVs combined treatment of lung cancer, which we retrieved from the clinical trial registry, is shown in the table below (**Table 1**). At the same time, we summarized the current clinical trials of OVs for lung cancer in **Supplementary Table 1** for readers’ reference and follow-up.

**Table 1:** Clinical trial of OVs combined therapy for lung cancer.

ClinicalTrials. gov ID	Phage	Patient type	OVs	Intervention/Treatment	Status
NCT 05076760	I	Solid tumors, first relapsed or refractory advanced/metastatic NSCLC	MEM-288	MEM-288 with nivolumab	Recruiting
NCT 06463665	II	NSCLC	Olvi-Vec (GLV-1h68)	Combination of Olvi-Vec followed by further platinum-based chemotherapy plus an ICI	Recruiting
NCT 03647163	I-II	Solid Tumors, refractory NSCLC or NEC	VSV-IFNβ-NIS	VSV-IFNβ-NIS in combination with pembrolizumab.	Recruiting
NCT 03740256	I	Advanced HER2 Positive Solid Tumors	Binary Oncolytic Adenovirus	Binary Oncolytic Adenovirus in Combination With HER2-Specific Autologous CAR T Cells	Recruiting
NCT 04725331	I/IIa	Metastatic/Advanced Solid Tumors	BT-001 (TG6030)	BT-001 with repeated IT administrations alone and in combination with IV infusions of pembrolizumab	Recruiting
NCT 02824965	Ib	Advanced NSCLC	CAVATAK™ (CVA21)	Pembrolizumab in combination with intravenous CVA21	unknow

## 6 Prospect and challenge of oncolytic virus combination therapy

Through the synergistic effects of multiple mechanisms of action, OVs combination therapy can more effectively kill tumor cells, suppress tumor growth and metastasis. This combination therapy can stimulate a powerful anti-tumor immune response in the body, not only destroying tumor cells directly, but also activating the immune system’s memory function, allowing the body to produce long-term immune surveillance against tumor cells. With the deepening study of OVs and the biological characteristics of lung cancer, doctors can now develop personalized treatment plans for patients based on factors such as the type of tumor, genetic characteristics, and immune status. This personalized treatment strategy can better adapt to individual differences in patients and improve the precision and effectiveness of treatment, providing better medical services for patients (42, 63). Currently, the most studied OVs in the field of lung cancer are Adenoviruses, Coxsackieviruses, Herpes viruses, Newcastle disease virus, Vesicular stomatitis virus (64). With the improvement of genetic technology, researchers have been able to perform more precise genetic modifications and modifications of oncolytic viruses to enhance their cytolytic efficiency and specificity, while reducing their side effects. It is expected that more OVs with genetically edited optimization will be studied in the future.

There are studies that combine oncolytic viruses with specific tumor antigens to prepare tumor vaccines (65, 66). Tumor vaccines are a new cancer treatment method. The principle of using OVs in tumor vaccine design is to introduce genes encoding tumor antigens into virus vectors and input them into the body to stimulate the immune system to produce an immune response against tumor antigens. Den J et al. studied and proved that oncolytic viruses could enhance the therapeutic effects of DC vaccines in various tumor-bearing mouse models (67). Currently, there are studies that propose the synergistic effects of personalized cancer vaccines and anti-PD therapy in the “cancer immune cycle”. Personalized cancer vaccines can promote dendritic cells and APCs, antigen presentation, and T cell activation and activation, but will lead to an increase in PD-1/PD-L1 in the TME. PD-1/PD-L1 elevation, in turn, will induce effective immune suppression, limiting T cell recognition and attack on cancer cells, thereby limiting the efficacy of personalized cancer vaccines. Anti-PD therapy can help overcome this immune suppression (68). This combined application can promote an effective cancer “cancer immune cycle” and promote tumor regression (69). In general, the application of OV tumor vaccines is more in line with the direction of personalized medicine, and new vaccine delivery technologies such as nanotechnology are expected to improve the delivery efficiency of tumor vaccines (70). As an adjuvant for personalized administration of anti-cancer vaccines, OV is used in combination with immunosuppressive drugs, which has great application potential in the field of tumor therapy.

However, the clinical application of oncolytic viruses still faces some problems. Currently, the main method of OV treatment is intratumoral injection and intravenous injection. Intratumor injection has good local effects on the direct injection site tumor, but its range of action is small, and it cannot eradicate all metastatic lesions except for the local injection of the tumor lesion. Moreover, intratumoral injection is a damaging clinical operation, and patients are not willing to accept it, limiting its clinical application. Intravenous injection has less damage than intratumoral injection and is easier to apply, and it can deliver the drug to the entire body. Giti Esmail Nia et al. studied that when the OV-loaded carrier cells accumulated in the lungs, the primary lung tumor lesion achieved good therapeutic effects after intravenous injection (71). However, the majority of advanced NSCLC and almost always SCLC are associated with aggressive metastasis, which is the main cause of high overall mortality in lung cancer. OV treatment is effective for the primary site, but it cannot achieve sufficient clinical efficacy for lung cancer with metastasis. Therefore, it is urgently needed to improve targeting of all metastatic sites and further optimize systemic (intravenous) OV application for lung cancer. Another factor limiting its therapeutic efficacy is the sensitivity of OVs to innate and adaptive immune system factors such as complement and viral antibodies. Complement proteins have been found to impair the cytolytic function of OVs (72). The systemic infection of viral particles has been proven to be unable to avoid the immune factors in the body fluids. One proposed deliver method to solve this is based on using the patient’s own cells as a carrier for OVs, known as Trojan horse delivery. These OVs carrier cells are injected throughout the body to protect the OV they carry from innate and adaptive immune responses, achieving a “stealth” effect that idealizes transport to the tumor bed, leading to effective tumor infection and lysis. Of course, a lot of research is still needed to realize this therapeutic vision.

In addition, there are still some challenges in the further application of oncolytic virus combination therapy in lung cancer. The first is optimal combination therapy and timing selection. The combination therapy of oncolytic viruses involves the combination of multiple treatment approaches. How to choose the optimal combination therapy regimen and timing is a complex issue. It requires a comprehensive consideration of the patient’s condition, physical condition, the biological characteristics of the tumor, and the interactions between various treatment methods. Although some studies have explored different combination therapy modalities, more clinical and preclinical studies are needed to clarify the optimal treatment combination and sequence. For example, by conducting large-scale clinical trials to compare the efficacy and safety of different combination therapy regimens in different lung cancer patient populations, a rational treatment pattern and standard for selecting the appropriate treatment timing can be established (42). In addition, the infection efficiency, replication ability and stability of the virus vector for lung cancer and its metastases also need to be further optimized. To address these issues, researchers can improve their tumor targeting by, for example, modifying the virus’s surface proteins to more specifically recognize markers on the surface of tumor cells (73). At the same time, new viral vectors and delivery systems are developed to improve the efficiency of viral infection and the uniformity of tumor tissue distribution (71). For example, the combination of OVs with nanotechnology allowing them to reach the tumor site more precisely (74, 75).

## 7 Conclusion

OVs combined treatment of lung cancer shows great potential and application prospect. Through its unique mechanism of action and synergistic effect with other therapeutic methods, it provides a new treatment option for lung cancer patients, which is expected to enhance the therapeutic effect and improve the prognosis. We believe that for patients with advanced lung cancer who have failed standard therapy or are refractory, OVs combination regimen can be tried more frequently in clinical practice, and OVs combined with ICI can be used as the first choice for the treatment of lung cancer after immune resistance. In the future, oncolytic virus combined therapy will play a greater role in the treatment of lung cancer, bringing new breakthroughs in the fight against lung cancer.
