# Supplementary material for: Combination therapy with oncolytic viruses for lung cancer treatment
Source: Front Oncol. 2025 Apr 3;15:1524079. doi: 10.3389/fonc.2025.1524079 (PMC12003109; doi:10.3389/fonc.2025.1524079)
Supplement: Supplementary file 1 [file Table1.docx]

Table1. the current clinical trials of OVs for lung cancer

| ClinicalTrials.gov ID | Intervention / Treatment | Conditions | Study Start | Current State | Phase | Reason for terminated |
| --- | --- | --- | --- | --- | --- | --- |
| NCT05205421 | Oncolytic Virus Injection(RT-01) | ES-SCLC | 2022-01-18 | UK | Ⅰ |  |
| NCT05886075 | Oncolytic Virus Injection (R130) | Relapsed/Refractory Advanced Solid Tumors | 2023-03-30 | UK | Ⅰ |  |
| NCT05860374 | Recombinant herpes simplex virus Ⅰ, R130 | Advanced solid tumors | 2023-03-02 | Recruiting | Early Phase Ⅰ |  |
| NCT05961111 | Recombinant herpes simplex virus Ⅰ, R130 | Advanced Solid Tumors | 2023-06-24 | Recruiting | Early Phase Ⅰ |  |
| NCT05180851 | Recombinant oncolytic adenovirus L-IFN injection（YSCH-01） |  | 2021-11-30 | UK | Early Phase Ⅰ |  |
| NCT06508307 | GC001 oncolytic virus injection | Advanced solid tumors | 2023-04-26 | Recruiting | Ⅰ |  |
| NCT05598268 | Biological: T3011 Herpes Virus | Advanced solid tumors | 2022-03-01 | UK | Phase Ⅰ/Ⅱa |  |
| NCT05788926 | TG6050,IV | Advanced NSCLC | 2023-04-05 | Recruiting | Ⅰ |  |
| NCT05076760 | MEM-288, Intratumoral Injection  Nivolumab | Solid tumors including NSCLC | 2022-02-23 | Recruiting | Ⅰ |  |
| NCT04725331 | BT-001, IT alone and in Combination With  Pembrolizumab,IV | Cutaneous or, Subcutaneous Lesions or Easily Injectable Lymph Nodes of Metastatic/Advanced Solid Tumors | 2021-02-25 | Recruiting | Ⅰ/Ⅱa |  |
| NCT03647163 | VSV-IFNβ-NIS in combination with pembrolizumab | Solid tumors | 2019-04-09 | Recruiting | Ⅰ/Ⅱa |  |
| NCT03740256 | CAdVEC, IT | Advanced HER2 Positive Solid Tumors | 2020-12-14 | Recruiting | Ⅰ |  |
| NCT05061537 | PF-07263689 alone or in combination with sasanlimab | selected locally advanced or metastatic solid tumors | 2022-10-14 | Terminated | Ⅰ | There are no changes to the risk-benefit for participants who have received PF-07263689 in the study. |
| NCT04301011 | TBio-6517,IT or IV and combined with pembrolizumab | Advanced solid tumors | 2023-01-23 | Terminated | Ⅰ/Ⅱa | UK |
| NCT04673942 | AdAPT-001,IT checkpoint inhibitor | Refractory Solid Tumors | 2021-03-29 | Recruiting | Ⅱa/Ⅱb |  |
| NCT04495153 | CAN-2409  ICI | stage III/IV NSCLC, on standard of care first line ICI treatment with evidence that the clinical response is inadequate | 2020-10-13 | Active, not recruiting | Ⅱ |  |
| NCT04695327 | Tumor Necrosis Factor Alpha and Interleukin-2 Coding Oncolytic Adenovirus (TILT-123) | Injectable Solid Tumors | 2021-01-11 | Recruiting | Ⅰ |  |

ES-SCLC: Extensive-Stage Small Cell Lung Cancer; TNBC: Triple negative breast cancer; NSCLC: Non-small cell lung cancer; ICI: immune checkpoint inhibitor; UK: unknown; IT: intratumoral injection; IV: Intravenous injection.
